# Sex-Dependent Vulnerability to *Cannabis* Abuse in Adolescence

**DOI:** 10.3389/fpsyt.2015.00056

**Published:** 2015-04-20

**Authors:** Tiziana Rubino, Daniela Parolaro

**Affiliations:** ^1^Department of Theoretical and Applied Sciences, and Neuroscience Center, University of Insubria, Busto Arsizio, Italy

**Keywords:** *Cannabis* abuse, adolescence, sex, human studies, animal models

## Abstract

The goal of this review is to summarize current evidence for sex differences in the response to cannabinoid compounds, focusing mainly on a specific age of exposure, i.e., adolescence. Preclinical as well as clinical studies are examined. Among the different possible underlying mechanisms, the consistent dimorphism in the endocannabinoid system and delta9-tetrahydrocannabinol metabolism may play a part. All the collected data point to the need of including females in basic research as well as of analyzing results for sex differences in epidemiological studies.

## Introduction

*Cannabis* continues to be the most widely used illicit substance among adolescents in the world, and more users are seeking treatment each year (1). Accumulating evidence suggests that exposure to *Cannabis* or its psychoactive ingredient delta9-tetrahydrocannabinol (THC) during the adolescent developmental window may act as a risk factor for the occurrence of psychiatric disorders later in life (2–4).

Despite the well-accepted notion that several neuropsychiatric disorders, such as depression, conduct problems, and autism, are sex-related [see, for review, Ref. (5–7)], very few papers have dealt with sex vulnerability to adolescent *Cannabis* abuse, both at the preclinical and clinical level. The main obstacle to this lies in the fact that research is still mainly focused on the male sex: male animals in preclinical research and male subjects in clinical studies. The potential sex influence is still routinely ignored or dismissed even when both sexes are included, as in some human studies where no sex-related analysis is performed, but all the subjects are regarded as “unisex.” Fortunately, the view that biological sex is unimportant in neuroscience is increasingly seen as a false assumption [see for a commentary Ref. (8)]. Notably, the National Institute of Health has recently asked the scientific community for sex and gender inclusion plans in preclinical research (9).

We hope from now on to witness an increasing amount of research considering both sexes. However, so far, few papers have dealt with the influence of this variable on the response to cannabinoids during adolescence. Most work has been done at the preclinical level, but some literature on humans is now also appearing. For the sake of accuracy, in this review we will take into account only papers where both male and females are considered, or papers applying exactly the same paradigm of exposure in male and female animals.

## Human Studies

Few studies exist on sex-dependent effects of adolescent *Cannabis* abuse in humans, so it is difficult to draw a precise picture of this phenomenon. Nonetheless, here we want to discuss some interesting observations. Generally, *Cannabis* use is more prevalent among males, who display an earlier age of onset of use and are more likely to be on a heavier use trajectory (10). As a consequence, males appear to be more likely than females to become dependent on *Cannabis* (11, 12). However, females tend to have shorter intervals between the onset of use and regular use or development of dependence (13, 14). Accordingly, females enter treatment for *Cannabis* use disorders after fewer years and less cumulative use compared to males (15). In general, *Cannabis* abuse is associated with a broad range of adverse health measures in both adolescent girls and boys (14). The existence of an overall sex-dependent effect has already been reported for other drugs of abuse (16), and specifically, female adolescent users seem to experience negative consequences of drug use earlier than male peers, and appear to be more likely to suffer from an internalizing disorder, such as depressive and anxiety disorders (16). Conversely, male substance abusers have more externalizing behaviors, such as aggressiveness and impulsivity (16). This seems to be true also for *Cannabis*. One of the first papers describing this correlation reported that daily *Cannabis* use was associated with a fivefold increase in anxiety and depression in young females, but not males (17). Accordingly, higher rates of comorbid mood and anxiety disorders in women have been recently observed in a large epidemiological study performed in the United States (18). Adolescent female abusers, who developed greater internalizing symptoms, exhibited larger right amygdala volumes relative to males and female controls (19). Interestingly, larger amygdala volumes were associated with increased depression and anxiety symptomatology (19). Similarly, Lai and Sitharthan (20) reported a significant association between *Cannabis* use disorder and mental health disorders, and again, higher comorbidity rates were observed for females. The most common mental disorders were major depression, personality disorder, schizophrenia, and severe stress disorder (20). Potential sex-differences have also been reported for *Cannabis* use and neurocognitive functioning (21). Specifically, *Cannabis* use was more consistently associated with poorer episodic memory performance in females and with poorer decision-making performance in males. Female *Cannabis* users presented a larger prefrontal cortex (PFC) volume compared to controls, whereas male users presented a smaller one (22). It is worth noting that among users, larger PFC total volume was associated with worse executive functioning, thus implying that females performed the worst. Finally, studying the association between *Cannabis* use and earlier age of onset of psychosis (AOP), researchers found that male users are the group with the earliest AOP. However, this seems to be independent of sex, and instead linked to the fact that males start first and consume more than females (23).

In conclusion, *Cannabis* abuse in humans appears to be associated with different responses in male and females, resembling what has already been seen with other drugs of abuse. The molecular bases of these sex differences need further investigation. Future studies should take into account the interaction between the endocannabinoid system and sex hormones, but also the fact that adolescent males and females undergo neuromaturation at separate rates, thus presenting differential trajectories of neuronal maturation at the same age (24, 25), that could hence be differently affected by *Cannabis*.

## Animal Studies

Animal models, although far from addressing the complexity of human disorders, allow experimental controls that are not possible in human studies. Moreover, they provide a valuable approach for the investigation of neurobiological substrates. Through this helpful tool, it has been confirmed that chronic administration of natural or synthetic cannabinoids during the adolescent period – using paradigms resembling heavy *Cannabis* abuse in humans – causes persistent behavioral alterations in adult animals [see, for review, Ref. (2, 4, 26)]. Cognition is one of the most explored brain functions after adolescent exposure to natural or synthetic cannabinoids. When sex was taken into account, it appeared that cannabinoid exposure during adolescence impaired learning and memory in both sexes. O’Shea et al. (27, 28) demonstrated that adolescent exposure to increasing doses of the synthetic cannabinoid agonist CP-55,940 for 21 days induced impaired recognition memory in the novel object recognition test long after discontinuation of the drug, in both female and male rats. However, when spatial memory was assessed in the Morris water maze test, adolescent cannabinoid exposure in both sexes disrupted learning immediately after the treatment (29), but not after a long drug-free period (29, 30). In the active place avoidance (APA) paradigm, where animal’s ability to learn and retrieve spatial information as well as flexibility of learning is assessed, early adolescent THC exposure did not affect the task acquisition, nor the performance after the 24-h retention interval in adult animals of both sexes (31). However, when flexibility was considered, impaired performance on the reversal trial of the APA task was observed (31). In the radial maze test, used to assess spatial working memory, both male and female rats showed deficits when tested long after adolescent exposure to THC (32, 33). These data suggest that adolescent exposure to cannabinoids induces long-term cognitive impairments specifically in recognition and spatial working memory, as well as in flexibility, whereas pure spatial memory does not seem to be affected. However, these effects do not display sex differences, since they are present in both male and female animals. Less consistent results have been obtained about the impact of adolescent cannabinoid treatment on anxiety behaviors. In fact, results coming from adult animals of both sexes exposed to cannabinoids during their adolescence showed all type of responses: anxiolytic-like response (34), anxiogenic-like effect (27, 28), or no changes in their behavior (35). Neither conclusions regarding the impact of adolescent exposure on anxiety behaviors nor about possible sex differences can be drawn from these findings. A different picture is present when the forced swim test was used: adolescent exposure to THC induced a significant increase in immobility that was apparent only in female rats (35, 36). Also, the effect of adolescent cannabinoid exposure on adult drug self-administration seems to present sex-dependency. Higher adult cocaine self-administration rates have been reported in female rats only (37), whereas increase in morphine self-administration under the fixed ratio 1 schedule has been described in males but not in females (38). As a whole, animal models seem to confirm the existence of some sex-dependent responses to adolescent cannabinoid exposure, with females appearing more sensitive than males in the emotional sphere.

These differences in behavior are substantiated by differences at the cellular/molecular level. Pharmacokinetics seems to play a part. It has been recently reported that adolescent female rats exhibit pronounced metabolism of THC to the still active compound 11-OH-THC compared to their male conspecifics, particularly after repeated THC administration (39). Thus, THC exposure could conceivably be potentiated by its active metabolite in female adolescents. This fact together with the observation that adolescent female rats possess more efficient CB1 receptors (40), suggests that they may be more vulnerable to THC effects. Accordingly, chronic THC exposure in adolescence induced more intense CB1 receptor desensitization in females, with more brain areas involved, despite similar down-regulation (35, 41). If confirmed also in humans, this would explain, at least in part, why females tend to have shorter intervals between the onset of use and the development of dependence, the so-called “telescoping effect” (13–15, 18). Another observation that comes from animal studies and deserves further investigation is that sex-dependent sensitivity appears to exist also with regard to the brain regions that are affected by the treatment. Specifically, in female animals, among all the cerebral areas investigated, the PFC seems to be the most affected, whereas it is the hippocampus in males. For example, Higuera-Matas et al. (30) reported that while periadolescent exposure to a fixed dose of a synthetic cannabinoid agonist did not produce robust behavioral effects, it did induce an increase of the plasticity marker PSA-NCAM in the hippocampus of males only. Similarly, Lee et al. (42) showed that a sustained adolescent CB1 receptor activation reduced adult hippocampal neurogenesis in both sexes; however, for some parameters, males appeared to be more greatly affected than females. Our group, in the search for a possible molecular correlate for the impaired spatial working memory induced by adolescent THC administration, investigated some markers of neuroplasticity in the PFC and hippocampus of both male and female rats (32, 33). Interestingly, a significant decrease in pre- and post-synaptic markers was present in the hippocampus of male rats, whereas the same proteins changed in the PFC of female animals (32, 33). Of note, in human *Cannabis* abusers, the occurrence of significant changes in the hippocampus of males (43) and in the PFC and amygdala of females (19, 22) have been observed. These brain regions are differently involved in the modulation of cognition (hippocampus and PFC) and emotion (amygdala and PFC), and this may explain the greater effect on emotionality in females.

## Conclusion

In conclusion, some sex-dependent effects exist in the response to cannabinoid compounds between adolescent males and females. These effects may rely on the different pharmacokinetics described for THC between males and females as well as on sex differences present in the endocannabinoid system. To complicate the picture, a fact that is specific for the adolescent population and should also be taken into account is represented by the observation that some brain developmental characteristics are different in the two sexes. For example, neurodevelopmental trajectories are significantly different between males and females [(25); see, for review, Ref. (44)]. Total brain size and regional gray matter volumes follow an inverted U shaped maturational curve and peak earlier in females, thus suggesting that the pruning process occurring in the adolescent brain might be present with different intensity in boys and girls of the same age. Since it has been recently suggested that the endocannabinoid system in the adolescent brain may play a part in synaptic pruning (45), exposure to cannabinoids during adolescence might differently interact with the pruning event in boys and girls, thus leading to different impairments in brain and behavior. Not least, interactions of the endocannabinoid system with gonadal hormones may also play a part. Interestingly, it has been recently suggested that sex hormones and the endocannabinoid system might work in symphony to promote maturational processes within the adolescent brain, specifically in those circuits important for the emotional and motivational response to sexually relevant stimuli (46). However, the existence of a close interaction between the endocannabinoid system and sex hormones has long been known. For example, CB1 receptor expression and density appear to be under the control of sex steroids in both males and females in some cerebral areas (47, 48). More recently, it has been reported that endocannabinoids and gonadal hormones may reciprocally regulate each other, and interestingly, estrogen can recruit endocannabinoids to modulate emotionality (49, 50). This is particularly important when considering that ovarian hormones may actively contribute to the remodeling event in the female brain during puberty and adolescence, as recently suggested by Juraska et al. (51). This was demonstrated for few brain areas; among them, there are the PFC and amygdala, the very same areas mainly affected by cannabinoids in adolescent females. A deeper knowledge of all these interactions would be helpful in designing proper sex-specific treatments or interventions to prevent or recover the long-term adverse effects induced by adolescent heavy *Cannabis* abuse.

## Conflict of Interest Statement

The authors declare that the research was conducted in the absence of any commercial or financial relationships that could be construed as a potential conflict of interest.
